# Habitat associations of day-flying Lepidoptera and their foodplants within nature reserves in Bedfordshire, UK

**DOI:** 10.1007/s10841-024-00554-7

**Published:** 2024-02-17

**Authors:** Esme Ashe-Jepson, Juliet Anderson, Gwen E. Hitchcock, Sky Wallis, Keira Wingader, Andrew J. Bladon, Edgar C. Turner

**Affiliations:** 1 Department of Zoology, University of Cambridge, Downing Street, Cambridge, CB2 3EJ UK; 2 The Wildlife Trust for Bedfordshire, Cambridgeshire and Northamptonshire, The Manor House, Broad Street, Cambourne, Cambridge, CB23 6DH UK; 3 Ecology Department, JBA Consulting, Epsom House, Red House Interchange, Doncaster, DN6 7FE UK; 4 Ecology and Evolutionary Biology Division, University of Reading, School of Biological Sciences, RG6 6EX Reading, UK

**Keywords:** Climate change, Butterfly, Lepidoptera, Larva, Habitat use, Habitat preference

## Abstract

**Abstract:**

Species often associate with specific habitat characteristics, resulting in patchy distributions, whereby they only occupy a proportion of available habitat. Understanding which characteristics species require is a valuable tool for informing conservation management. We investigated the associations of eleven species of day-flying Lepidoptera larvae and their foodplants with habitat characteristics within calcareous grassland reserves in Bedfordshire, UK, across two scales relevant to land managers and target species: the reserve (cardinal aspect, vegetation type) and foodplant patch scale (foodplant height and density). We investigated whether ecological traits (habitat specialism, as defined at a national-scale, and overwintering life stage) influenced the strength of associations. At the reserve scale, we found variation in associations with habitat characteristics across species, with species that overwinter at non-adult life stages having more restricted associations, indicating that they may be more vulnerable to environmental change. Associations were generally stronger with vegetation type than aspect, which can be manipulated more easily by land managers. Seven species had similar associations with habitat characteristics to their foodplants, implying that management to benefit foodplants will also benefit larvae. However, the remaining four species had different associations to their foodplants, and may require alternative management approaches. At the foodplant patch scale, four species were associated with foodplant characteristics, which could be used to inform effective fine-scale management.

**Implications for insect conservation:**

Implications for insect conservation: Diverse habitat associations imply that topographic and vegetation variation are valuable for supporting diverse assemblages of butterflies and their foodplants.

**Supplementary Information:**

The online version contains supplementary material available at 10.1007/s10841-024-00554-7.

## Introduction

Species often display patchy or uneven distributions, which can reflect associations with heterogenous components of an ecosystem. These include both biotic factors, such as the presence of hosts, prey, predators, or competitors (Wisz et al. 2012), or abiotic factors, such as the presence of suitable microclimates for thermoregulation (Eilers et al. 2013). Therefore, the drivers of species’ distributions or associations can be complex, and interact with each other in ways that result in only a small fraction of the landscape being suitable for each species (e.g., Lane et al. 2001). Understanding which environmental resources species associate with can be important for informing and prioritising conservation of both species and landscapes (Cañadas et al. 2005).

Many species are losing suitable habitat due to anthropogenic change (Prakash & Verma 2022). In terrestrial systems, land-use change is the largest driver of biodiversity loss (Jaureguiberry et al. 2022). The multi-faceted changes occurring during the Anthropocene can also amplify other drivers of biodiversity decline. For example, species that have suffered from habitat loss may be more affected by climate change, due to an inability to disperse and track suitable climate (Oliver et al. 2017). Similarly, species can be buffered from the effects of a changing climate if they are able to use habitat heterogeneity and thermal refugia within a landscape (Stark and Fridley 2022). Therefore, there is a growing need to understand species’ associations with habitat characteristics and specific resource requirements, to predict the long-term effects of change and to develop management approaches that minimise the impacts of interacting anthropogenic drivers. However, studies on habitat association tend to be focussed on single species (e.g. Vehanen et al. 2003; Davies et al. 2006; Hayes et al. 2018), and relatively little is known about broad-scale habitat associations across communities. As requirements can vary between individual species within a habitat, optimal management options may differ within communities, making it hard to determine the best management decision from species-specific studies (Slamova et al. 2013). As such, it is important to consider patterns of associations with habitat characteristics across species, to determine how land management will affect different species within a community, and to identify which management approach confers the greatest benefits for long-term biodiversity conservation.

Lepidopterans make an excellent model group to investigate associations with habitat characteristics, as they represent one of the largest and most diverse groups of organisms on Earth (Whiting 2002). As holometabolous insects, they have complex life cycles, where life stages differ in morphology, behaviour, ecology, and resource requirements (MacLean et al. 2016). Therefore, different species are likely to associate with different habitat characteristics. As with most holometabolous insects, adult Lepidoptera tend to be highly mobile, and able to search a relatively large area for resources (Doak et al. 2006). In comparison, larvae are relatively immobile, often being restricted to their foodplant and the immediate surroundings (Dethier 1959; Weiss et al. 1987). This limited dispersal ability may mean larvae are under strong constraints to occupy habitats that maximise their fitness, and may have more restricted associations with habitat characteristics than adults. Larval habitat associations tend to reflect where adult females oviposit, and selected foodplants often represent only a small proportion of those available (Dethier 1959; Salgado et al. 2020; Ashe-Jepson et al. 2022). The ultimate drivers behind these associations are poorly understood (Singer 2004), but are likely to reflect habitat characteristics that benefit larval fitness (Doak et al. 2006; Anthes et al. 2008).

A key driver of larval distribution is the distribution of foodplants (Quinn et al. 1998), and strong habitat associations of the foodplant may obscure larval associations with underlying habitat characteristics. As such, it is important to understand the habitat requirements and distributions of foodplants in combination with larval distribution. Where foodplant and larval associations differ, this may result in only a small proportion of available foodplants being suitable for Lepidoptera, placing high pressure on this fraction of suitable habitat. Despite the importance of understanding the habitat associations of larvae and their foodplants, studies that incorporate both are rare. By disentangling whether larval distributions are driven by foodplants or other aspects of the environment, land managers and conservation practitioners can develop more effective land management strategies.

Lepidoptera benefit from protected land. In Germany, butterfly species richness is higher within protected areas than in surrounding unprotected land (Rada et al. 2019), and in the UK, climate-induced range shifts have resulted in increased colonisation of protected areas by butterfly species (Thomas et al. 2012). However, these benefits can be restricted by inappropriate land management. For example, in the Czech Republic, inappropriate grassland management resulted in the local extinction of the threatened *Colias myrmidone* (Konvicka et al. 2008). Of the 28% of protected land in the UK, only 11.4% is designated primarily for nature conservation. Within this, only 43–51% of protected areas were found to be in good condition and meeting conservation objectives, implying that as little as 4.9% of land in the UK is protected and effectively managed for nature (see Starnes et al. 2021 for full details of how these values were calculated). With such small areas protected, there is enormous pressure to maximise biodiversity within them. In the UK, 80% of butterfly species have declined in abundance or distribution since the 1970s (Fox et al. 2023) and there is substantial variation between species in their ecology, including habitat specialism, diet breadth, and overwintering life stage (Asher et al. 2001). This makes British Lepidoptera a particularly valuable group for studying factors affecting associations with habitat characteristics. Improving understanding of larval and foodplant associations, and which ecological traits influence this, could help to identify areas most valuable for species conservation, species most vulnerable to change, and the most effective land management practices to conserve threatened species.

Lepidopteran associations with habitat characteristics occur at different scales. For example, at the nature reserve scale, associations with topographic aspect or vegetation type may reflect thermal requirements (Eilers et al. 2013). Alternatively, associations at the foodplant scale may reflect specific characteristics that benefit larval survival or growth, such as providing more food resources for development (Anthes et al. 2008). For example, *Cupido minimus* egg-laying females prefer taller, more apparent foodplants for oviposition, presumably as these larger foodplants are more detectable and/or provide more larval resources (Ashe-Jepson et al. 2022). The reserve and foodplant patch scale are particularly relevant for land managers, who can select and prioritise land with certain aspects, or manage for vegetation types and foodplant characteristics, to provide the right resources for different butterfly species.

In this study, we focus on an assemblage of 11 Lepidoptera species, located across a network of four chalk grassland reserves, to address the following questions:

Are larval Lepidoptera and their foodplants associated with habitat characteristics at the reserve scale, including topographic aspect and vegetation type?
  We hypothesise that larval Lepidoptera and their foodplants will be associated with reserve scale habitat characteristics, due to differing thermal and moisture characteristics between different areas. Previous studies have identified Lepidoptera associations with aspect (Davies et al. 2006; Anthes et al. 2008), and vegetation type (Suggitt et al. 2012).
Are larval Lepidoptera associated with habitat characteristics at the foodplant patch scale, including plant height and foodplant density?
  We hypothesise that Lepidoptera will have associations with foodplant height and density, as these are likely to correspond to more resources for larvae. Previous studies have identified associations of lepidopteran larvae with both foodplant height (Valdés & Ehrlén 2018; Ashe-Jepson et al. 2022) and density (Batáry et al. 2007).
3. Does the strength of association with habitat characteristics at the reserve scale and foodplant patch scale differ between Lepidoptera with different ecological traits, including habitat specialism [as defined at a national scale (Asher et al. 2001)] and overwintering life stage, and are these patterns consistent across scales?

We hypothesise that species considered to be habitat specialists will display stronger associations with habitat characteristics than generalists across both scales. We hypothesise that species that overwinter as less mobile life stages (egg, larva, pupa) will have stronger associations at the reserve scale than species that overwinter as adults, as they must occupy an area for a longer period of time during a vulnerable period of their life cycle. These ecological traits were selected as they are particularly relevant to the larval life stage. At the foodplant patch scale, we hypothesise that overwintering life stage should not influence the strength of association with specific foodplant characteristics, as these characteristics could change across seasons and are therefore less valuable as predictors of overwintering site quality.

## Methods

### Study sites

**Fig. 1:**
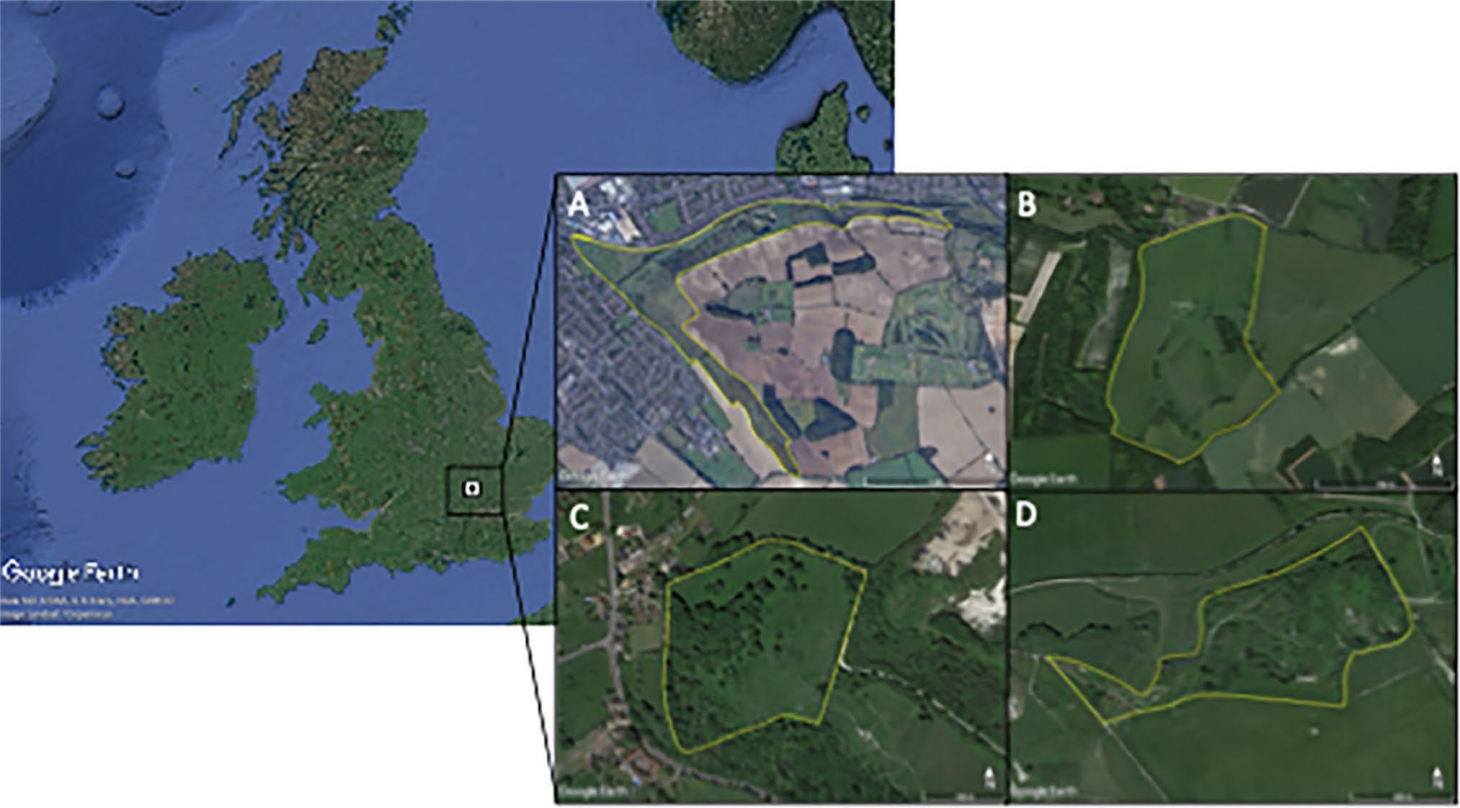
Study site locations in the UK (inset) and in Bedfordshire. The white square marks the location of the four study sites, which are also outlined in yellow. **A** Blow’s Downs (51°52′47.57″ N, 0°29′10.38″ W), **B** Pegsdon Hills (51°57′13.94″ N, 0°22′06.98″ W), **C** Totternhoe Knolls (51°53′22.78″ N, 0°34′49.81″ W), **D** Totternhoe Quarry (51°53′30.75″ N, 0°34′09.37″ W). (Color figure online) *Source* Google Earth Pro v.7.3.3.7786, 51°54′42.85″ N, 0°28′49.36″ W, eye alt 72.0 km. Data SIO, NOAA, US Navy, NGA, GEBCO. Image Landsat/Copernicus. © Google Earth. Imagery date: 13/06/2021 (accessed 13 February 2023). (Color figure online)

Lepidoptera were sampled across four sites in Bedfordshire, UK, all managed by the Wildlife Trust for Bedfordshire, Cambridgeshire and Northamptonshire (WTBCN; Wildlife Trust for Bedfordshire, Cambridgeshire, and Northamptonshire 2023): Blow’s Downs (51°52′47.57″ N, 0°29′10.38″ W), Pegsdon Hills (51°57′13.94″ N, 0°22′06.98″ W), Totternhoe Knolls (51°53′22.78″ N, 0°34′49.81″ W), and Totternhoe Quarry (51°53′30.75″ N, 0°34′09.37″ W) (Fig. 1). All sites are calcareous grassland reserves with highly variable topography, largely the result of medieval quarrying, and are composed of a mixture of exposed chalk, short grass, long grass, and scrub.

Eleven species of Lepidoptera were selected for surveys. These were chosen because they are known to be abundant and to breed within the nature reserves, because they encapsulate variation across the ecological traits of interest, and because they include species that are considered to be specialist and generalist at the national scale (Tables 1, S1). Habitat specialism categories follows Asher et al. (2001), where habitat generalists are characterised as species with broad habitat requirements, use habitats that are widely distributed, and can use linear habitats such as hedgerows and road verges. Habitat specialists are defined as being restricted to discrete patches of habitats that are localised or patchy in the modern landscape, and rarely or never use linear habitats. The categories assigned represent the characteristics displayed across the majority of the species range.

**Table 1:** The 11 Lepidoptera species sampled, the nature reserves they were surveyed on (BD = Blow’s Downs, PH = Pegsdon Hills, TK = Totternhoe Knolls, TQ = Totternhoe Quarry, or All if all four reserves were surveyed); the survey method used (Focused = focused searches, Suction = suction sampling); which foodplant species were surveyed; how foodplants were searched (‘All’ = all foodplants in that reserve were searched, ‘Random plots’ = the foodplant was too abundant to search all areas and so the foodplant was mapped within 45 random 5 m^2^ plots); whether the foodplant density within 30 cm of an occupied plant was recorded by ‘Count’ (count of individuals; in cases where foodplants are distinct and non-overlapping), or by ‘Percent’ (percentage cover; where foodplants are non-distinct or creeping); which life stage was searched for (egg or larva); the sample size achieved (number of Lepidoptera records across all foodplants); and the months and years the surveys took place

Species	Family	Reserves	Survey method	Foodplant species	Foodplants searched	Foodplant density	Life stage	Sample size	Months surveyed	Years surveyed
*Erynnis tages*	Hesperiidae	TQ	Focused	*Lotus corniculatus*	Random plots	Percent	Egg	31	June-July	2021, 2022
*Cupido minimus*	Lycaenidae	TQ	Focused	*Anthyllis vulneraria*	All	Count	Egg	45	July	2020
*Polyommatus coridon*	Lycaenidae	TQ	Focused	*Hippocrepis comosa*	Random plots	Percent	Larva	43	June	2021
*Aglais io*	Nymphalidae	All	Focused	*Urtica dioica*	All	Percent	Larva	274	June-July	2021, 2022
*Aglais urticae*	Nymphalidae	All	Focused	*Urtica dioica*	All	Percent	Larva	104	June-July	2021, 2022
*Maniola jurtina*	Nymphalidae	TQ	Suction	Grasses	Random plots	NA	Larva	25	May	2021, 2022
*Vanessa atalanta*	Nymphalidae	All	Focused	*Urtica dioica*	All	Percent	Larva	24	June-July	2021, 2022
*Anthocharis cardamines*	Pieridae	BD, PH	Focused	*Alliaria petiolata*	All	Count	Egg	137	May–June	2021
*Gonepteryx rhamni*	Pieridae	TQ, TK	Focused	*Rhamnus cathartica*	All	Count	Larva	83	June	2022
*Pieris napi*	Pieridae	BD	Focused	*Alliaria petiolata*	All	Count	Egg	34	July	2021, 2022
*Zygaena filipendulae*	Zygaenidae	TQ	Suction	*Lotus corniculatus*	Random plots	Percent	Larva	13	May	2021, 2022

Survey months were selected to capture the peak abundance of either eggs or larvae

### Lepidoptera foodplant surveys at the reserve scale

Initial surveys focused on locating Lepidoptera larval foodplants in the reserves where foodplants and associated Lepidoptera were present. In cases where the foodplant was not abundant, we searched the whole reserve, across all vegetation types except in dense scrub, getting within 10 m of all locations, and recorded all foodplant patches present. To be considered a patch, foodplants had to be within 5 m of each other and to share the same aspect and vegetation type. Foodplant patches were marked with a GPS point, and environmental characteristics in the surrounding 5 × 5 m were recorded. These were aspect (North, East, South or West for slopes > 10°, and Flat where slopes were < 10°), and vegetation type (short grass (grass < 10 cm in average height), long grass (grass > 10 cm in average height), or encroaching scrub (25–75% of the surrounding 5 m^2^ area covered by scrub).

Where foodplants were too abundant to survey across the entire reserve, stratified random points were generated to represent three replicates of each combination of aspect (North, East, South, West, Flat) and vegetation type (short grass, long grass, encroaching scrub), resulting in 45 randomly selected locations per reserve, where we established 5 × 5 m plots. These plots were searched by hand for foodplants (by count if the foodplants were distinct and non-overlapping, or by percentage cover for creeping foodplants). We also recorded average vegetation height in the centre of each plot, by gently lowering an A4 clipboard onto the surrounding vegetation until it rested at an average height, which was then measured from the ground with a tape measure. This ensured that single tall stems of vegetation did not have an undue influence on our height measures.

### Lepidoptera surveys and foodplant scale characteristics

At the appropriate time of year to coincide with the peak abundance of each target species, foodplants that had been located earlier were surveyed using one of two methods, depending on the foodplant (Table 1). Multivoltine butterfly species were surveyed within a single brood, with the brood with the highest abundance being selected (see Table 1 for dates surveyed).

Grass-feeding species were sampled in the 45 random plots using a suction sampler (Mountfield MBL 270 V 27.6CC 2-stroke petrol blower and vacuum (airflow speed = 161 mph), modified to have a fine mesh net in the vacuum intake to catch invertebrates, on vacuum setting). The suction sampler was used at full power for three seconds every 1 m^2^ (resulting in 25 suction samples within each of the random plots, that were subsequently pooled), with the net emptied every 5 suction samples to prevent over-filling. Captured larvae were identified to species, and released after recording.

For non-grass feeding species, all located foodplants (either within the 45 random plots, or across the reserve) were searched individually by hand for eggs or larvae (depending on which was more conspicuous; egg counts were used as proxies for larval counts and hereafter are referred to as larvae for simplicity) (Table 1). Once a larva was found, foodplant scale characteristics were recorded: vegetation height (using the clipboard method described above), foodplant height (using a tape measure from the ground to the tallest part of the plant), and foodplant density (for individual plants this was a count of foodplants within a 30-cm radius, and for creeping or non-distinct foodplants was an estimate of percentage cover within a 30-cm radius by eye). Where the foodplant was over 2 m high (*Rhamnus cathartica*, the foodplant of *Gonepteryx rhamni*), the height was estimated by eye by a single recorder. Control foodplants were selected by identifying a point 1 m from the focal foodplant in each sequential cardinal direction (North, East, South and West, in rotation), and selecting the closest unoccupied foodplant to that point.

### Statistical analyses

All analysis took place in R version 3.6.1 (R Core Development Team, http://www.r-project.org). Plots were produced using the ‘ggplot2’ package (Wickham 2016).

### Reserve scale habitat associations

To test whether foodplant or larval distribution was associated with habitat characteristics (cardinal aspect or vegetation type, separately) at the reserve scale, foodplant and larval counts were summed by habitat across years and sites. Foodplant and larval counts in each habitat characteristic were tested separately with chi square tests, using the proportional area of each characteristic present at the sampled site(s) to generate expected frequencies. Monte Carlo simulations were used with 10,000 replicates and simulated p values to account for the small sample size of some counts.

Three tests were performed for each plant and Lepidoptera species (except for the grass-feeding species, *M. jurtina*). Firstly, counts of foodplants across habitat characteristics were tested against the proportional area available of each habitat characteristic (‘Foodplant’). Strong associations here would demonstrate that foodplants are disproportionately found on particular aspects or vegetation types. Secondly, counts of larvae across habitat characteristics were tested against the proportional area of each habitat characteristic present (‘Larva’). Strong associations here would demonstrate that larvae are disproportionately found on particular aspects or vegetation types. Thirdly, counts of larvae in each habitat characteristic were tested against the proportional distributions of their associated foodplant in each habitat characteristic (‘Larva * foodplant’). Strong associations here would demonstrate that larval associations are independent from their foodplant’s associations, while weak associations would demonstrate that the distribution of larvae is following the distribution of their foodplant. For each analysis, Cramer’s V was calculated to quantify the strength of association (Akoglu 2018), using the ‘lsr’ package in R (Navarro 2015). Cramer’s V can vary between 0 (no association) and 1 (perfect association). By comparing the strength of association between these categories, we can identify whether the larval distribution is primarily determined by their own association with a particular habitat (little change between ‘Larva’ and ‘Larva * foodplant’), or whether larval distribution is more strongly driven by association with the foodplant (weakening of association from ‘Larva’ to ‘Larva * foodplant’).

### Foodplant scale habitat associations

To test whether eggs or larvae were associated with habitat characteristics at the foodplant scale, conditional logistic regressions were fitted for each species using the ‘survival’ package in R (Therneau 2022), with presence/absence as the response variable, and foodplant height and density as explanatory variables. Model assumptions were checked before fitting. Due to generally high correlations with foodplant height, vegetation height was excluded from analyses (Table S2, Dormann et al. 2013). Data were stratified into pairs (focal plant with larva present and control plant in the same patch with no larvae). The two species sampled with suction samplers (*M. jurtina*, *Z. filipendulae*) were excluded from this analysis as it was not possible to determine which specific foodplant they occupied before capture.

### Habitat associations in relation to ecological traits

To determine whether the ecological traits of species influence the strength of associations with habitat characteristics (Cramer’s V at the reserve scale, exponentiated coefficients at the foodplant patch scale), species were grouped according to their traits [habitat specialism (specialists and generalists at the national scale), and overwintering life stage (adult and non-adult)] (Table S1), and the data were tested using Wilcoxon Rank Sum tests separately between the scales. This test was selected due to the response variables (strength of association) being non-normal. The data were then visually inspected to identify whether patterns were consistent across scales.

## Results

### Reserve scale habitat associations

**Fig. 2:**
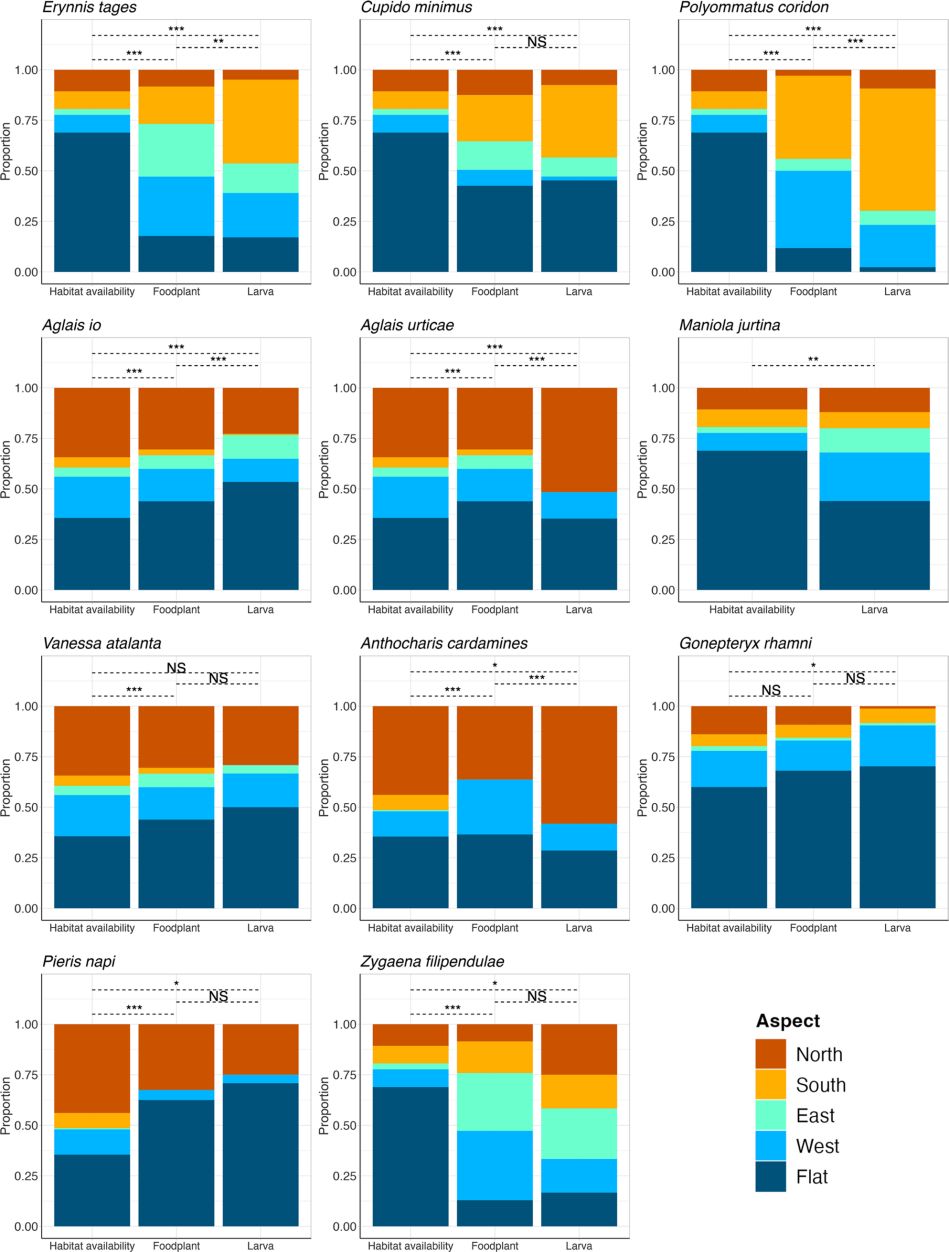
The proportional availability of each habitat characteristic across cardinal aspects (North, South, East, West, and Flat, where the slope was < 10°) and associated proportional distributions of foodplants and Lepidoptera larvae across all years and study sites. Significant associations between habitat availability, foodplant, and Lepidoptera distributions are shown with dashed lines above the bars, and are denoted with NS = non-significant, *p < 0.05, **p < 0.01, ***p < 0.001. Species are ordered alphabetically by family and species. Foodplant species are as follows: *Erynnis tages* with *Lotus corniculatus*; *Cupido minimus* with *Anthyllis vulneraria*; *Polyommatus coridon* with *Hippocrepis comosa*; *Aglais io* with *Urtica dioica*; *Aglais urticae* with *Urtica dioica*; *Maniola jurtina* with grasses; *Vanessa atalanta* with *Urtica dioica*; *Anthocharis cardamines* with *Alliaria petiolate*; *Gonepteryx rhamni* with *Rhamnus cathartica*; *Pieris napi* with *Alliaria petiolate*; *Zygaena filipendulae* with *Lotus corniculatus*

**Fig. 3:**
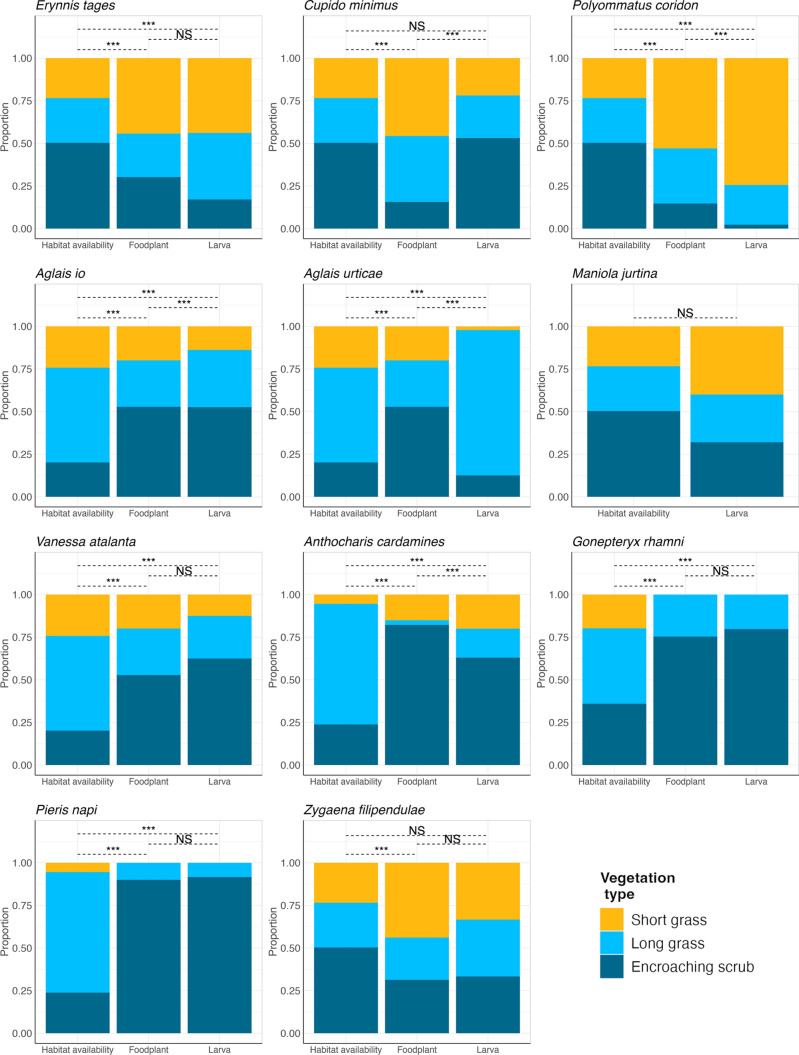
The proportional availability of each habitat characteristic across vegetation types (SG = short grass (average height < 10 cm), LG = long grass (average height > 10 cm), ES = encroaching scrub (25–75% of the surrounding 5 m^2^ contains scrub), and associated proportional distributions of foodplants and Lepidoptera larvae across all years and study sites. Significant associations between habitat availability, foodplant, and Lepidoptera distributions are shown with dashed lines above the bars, and are denoted with NS = non-significant, *p < 0.05, **p < 0.01, ***p < 0.001. Species are ordered alphabetically by family and species. Foodplant species are as follows: *Erynnis tages* with *Lotus corniculatus*; *Cupido minimus* with *Anthyllis vulneraria*; *Polyommatus coridon* with *Hippocrepis comosa*; *Aglais io* with *Urtica dioica*; *Aglais urticae* with *Urtica dioica*; *Maniola jurtina* with grasses; *Vanessa atalanta* with *Urtica dioica*; *Anthocharis cardamines* with *Alliaria petiolate*; *Gonepteryx rhamni* with *Rhamnus cathartica*; *Pieris napi* with *Alliaria petiolate*; *Zygaena filipendulae* with *Lotus corniculatus*

**Fig. 4:**
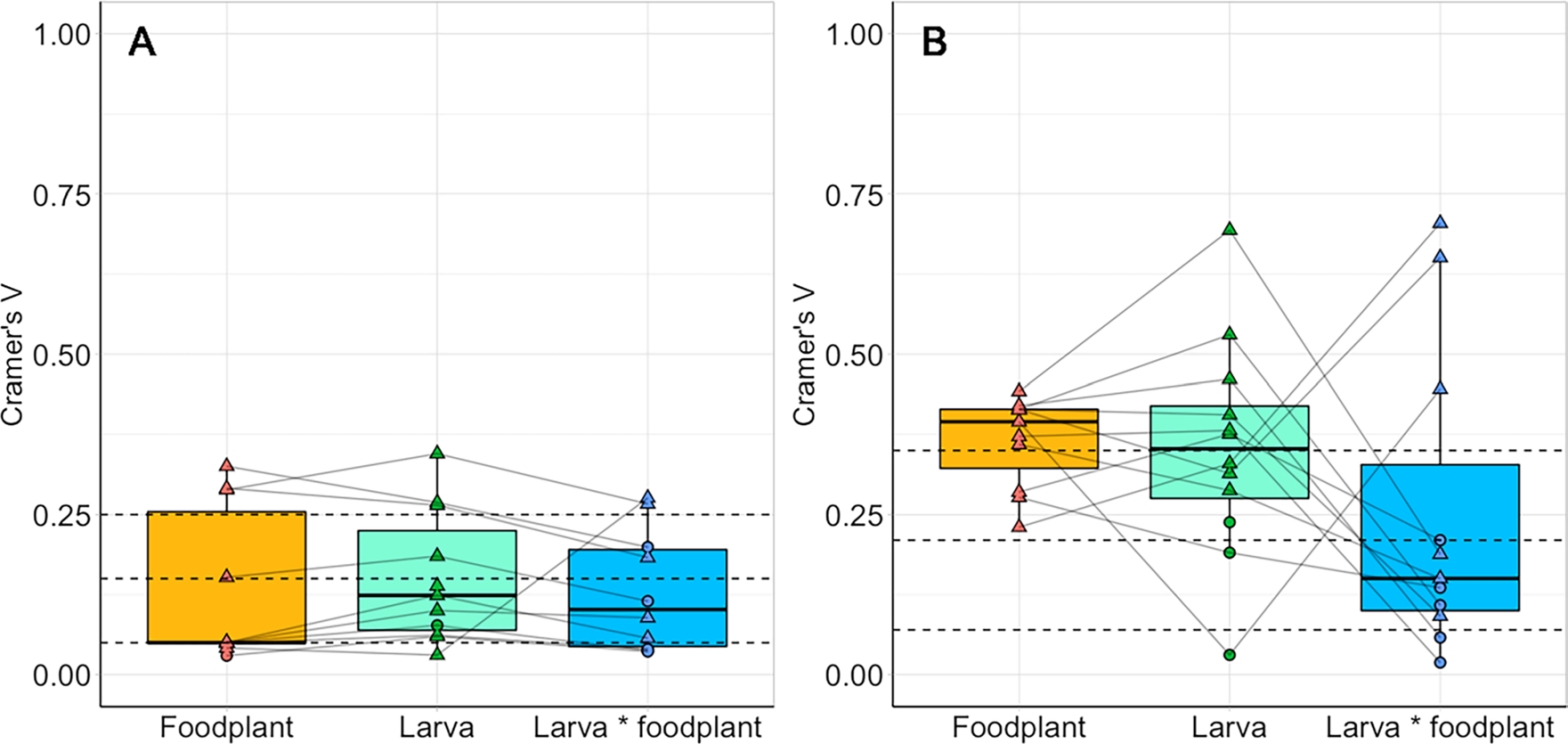
The strength of association (Cramer’s V) of foodplants and larvae against the total available habitat area (Foodplant and Larva), and of larvae against the foodplant distribution (Larva * foodplant), across all eleven species for **A** cardinal aspect, and **B** vegetation type. Horizontal dashed lines indicate weak, moderate, and strong associations to aid interpretation, calculated from the degrees of freedom. All data points are shown; significant values are denoted with triangles, non-significant values are denoted with circles. Lines connect datapoints within foodplant–Lepidoptera species pairs

Associations with habitat characteristics (aspect and vegetation type) at the reserve scale varied across foodplant and Lepidoptera species (Table 2, S3, Figs. 2, 3). Across all species, associations tended to be stronger with vegetation type than aspect (Fig. 4). Foodplant associations with aspect ranged from no association (*R. cathartica*) to strong associations (*Lotus corniculatus,* Cramer’s V = 0.325) (Fig. 2). In contrast, all foodplants had significant associations with vegetation types, ranging in strength from moderate (*L. corniculatus*, Cramer’s V = 0.277) to strong (*Hippocrepis comosa*, Cramer’s V = 0.442) (Fig. 3). Larval associations with aspect ranged from no association (*V. atalanta*) to strong (*P. coridon,* Cramer’s V = 0.345) (Fig. 2). Similarly, larval associations with vegetation type ranged from no association (*C. minimus*, *Z. filipendulae*) to strong (*P. coridon,* Cramer’s V = 0.693) (Fig. 3).

**Table 2:** The results of habitat associations at the reserve scale (cardinal aspect and vegetation type) and at the foodplant scale [plant height (cm), plant density (count of plants or percentage cover within 30 cm)]

Lepidoptera species	Ecological traits		Reserve scale						Foodplant scale			
			Aspect			Vegetation type			Plant height		Plant density	
	Specialism	Over-wintering stage	P value	Cramer's V	Association	P-value	Cramer's V	Association	P value	Probability	P value	Probability
*Erynnis tages*	Specialist	Larva	**0.004**	0.183	South	NS	*0.21*	NA	NS	- 19.5%	NS	+ 2.9%
*Cupido minimus*	Specialist	Larva	NS	*0.115*	NA	** < 0.001**	**0.446**	Encroaching scrub	**0.025**	** + 8.9%**	NS	+ 7.5%
*Polyommatus coridon*	Specialist	Egg	** < 0.001**	**0.267**	South	** < 0.001**	*0.188*	Short grass	NS	+ 11.4%	NS	- 5.2%
*Aglais io*	Generalist	Adult	** < 0.001**	*0.057*	Flat	** < 0.001**	*0.092*	Encroaching scrub	NS	+ 0.8%	** < 0.001**	**0.046**
*Aglais urticae*	Generalist	Adult	** < 0.001**	*0.089*	North	** < 0.001**	**0.651**	Long grass	0.054	+ 5.4%	**0.015**	**0.038**
*Maniola jurtina*	Generalist	Larva	**0.008**	*0.139*	Flat	NS	0.238	NA	NA	NA	NA	NA
*Vanessa atalanta*	Migrant	Adult	NS	*0.038*	NA	NS	*0.109*	NA	NS	+ 6.9%	NS	+ 3.4%
*Anthocharis cardamines*	Generalist	Pupa	** < 0.001**	**0.276**	North	** < 0.001**	*0.15*	Encroaching scrub	** < 0.001**	** + 3.2%**	NS	NA
*Gonepteryx rhamni*	Generalist	Adult	NS	*0.036*	NA	NS	*0.058*	NA	NS	- 1.1%	NS	+ 0.00%
*Pieris napi*	Generalist	Pupa	NS	*0.04*	NA	NS	*0.019*	NA	NS	+ 4.5%	NS	+ 9.3%
*Zygaena filipendulae*	Generalist	Larva	NS	0.199	NA	NS	*0.136*	NA	NA	NA	NA	NA

Reserve scale values represent Larva * Foodplant values, see Table S3 for all values. P-values are given for significant associations (in bold), non-significant p-values are denoted with ‘NS’. At the reserve scale, strength of associations are given [Cramer’s V, calculated from the degrees of freedom, ranging from 0 (low) to 1 (high)], and cells are shaded by this value, with italic and underlined values indicating a weak association and bold underlined values indicating a strong association. At the foodplant scale, probability values indicate changes in probability of presence with one unit increase per variable. Ecological traits of species are also listed (habitat specialism: generalist, specialist, or migrant; and overwintering life stage: egg, larva, pupa, or adult), from Asher et al. (2001). Species are listed alphabetically by family and species.

Two Lepidoptera species were associated with south-facing slopes (*E. tages*, *P. coridon*), two with north-facing slopes (*A. urticae*, *A. cardamines*), and two with flat ground (*A. io*, *M. jurtina*) (Fig. 2). Three species showed associations with encroaching scrub (*C. minimus*, *A. io*, *A. cardamines*), one with long grass (*A. urticae*), and one with short grass (*P. coridon*) (Fig. 3, Table 2).

### Foodplant scale habitat associations

The likelihood of larval presence on a foodplant in response to foodplant height and density varied between species (Table S4). Five species showed no association with the measured foodplant characteristics (*E. tages,* χ^2^ = 1.03, d.f. = 2, p = 0.596; *P. coridon,* χ^2^ = 3.75, d.f. = 2, p = 0.154; *V. atalanta,* χ^2^ = 5.29, d.f. = 2, p = 0.071; *G. rhamni*, χ^2^ = 2.38, d.f. = 2, p = 0.305; *P. napi*, χ^2^ = 0.66, d.f. = 2, p = 0.718). Four species had significant associations with foodplant characteristics (*C. minimus*, χ^2^ = 10.93, d.f. = 2, p = 0.004; *A. io*, χ^2^ = 33.15, d.f. = 2, p < 0.001, *A. urticae,* χ^2^ = 11.55, d.f. = 2, p = 0.003; *A. cardamines*, χ^2^ = 21.62, d.f. = 2, p < 0.001). Of these, two species were more likely to be found on taller foodplants (*C. minimus*, z = 2.24, p = 0.025; *A. cardamines*, z = 3.95, p < 0.001), while two species were more likely to be found on foodplants in denser patches (*A. io,* z = 4.00, p < 0.001; *A. urticae,* z = 2.42, p = 0.015) (Table 2).

### Habitat associations across ecological traits

**Fig. 5:**
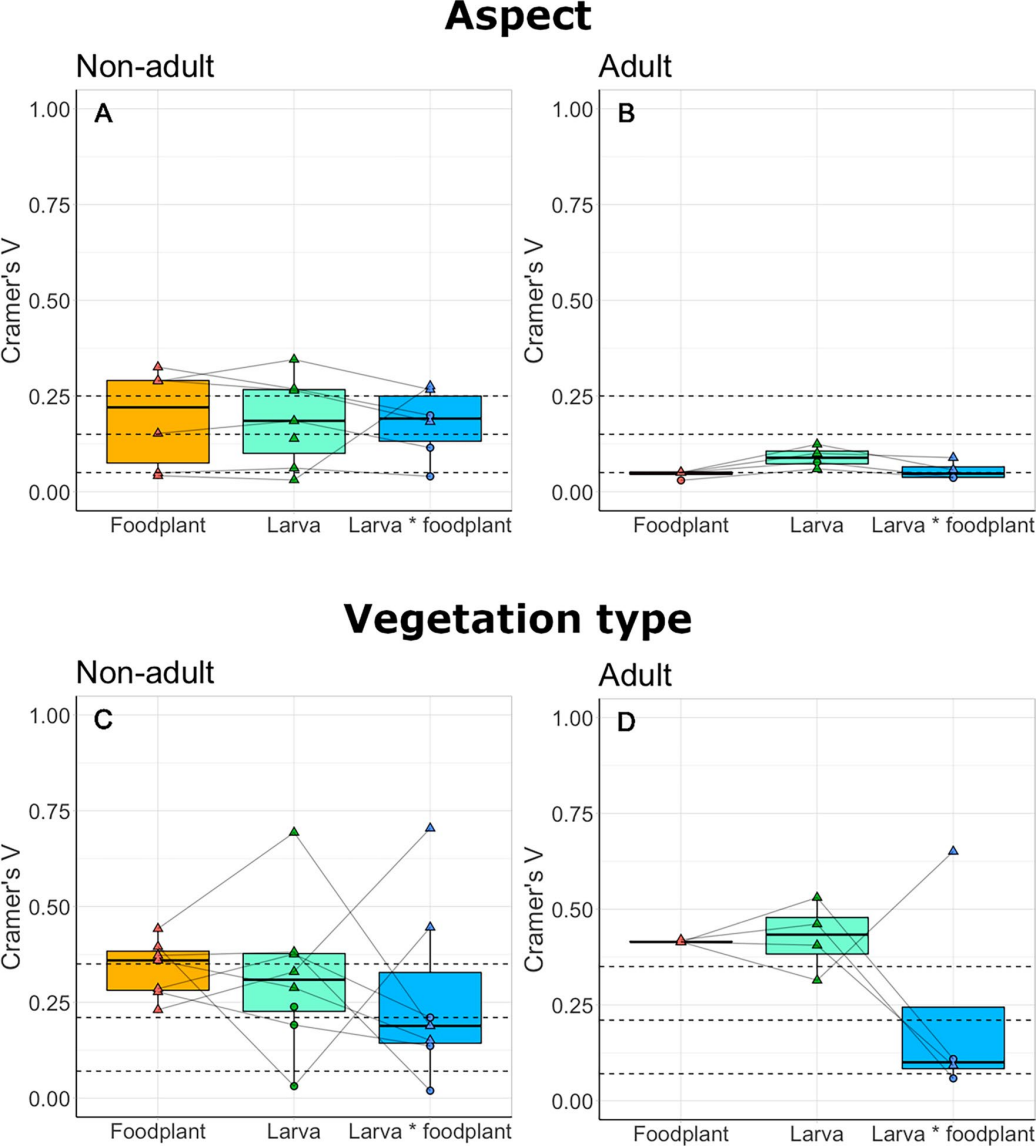
The strength of association (Cramer’s V) of foodplants (Foodplant) and larvae (Larva) against the total available habitat area, and larvae against the foodplant distribution (Larva * foodplant), between species that overwinter as a non-adult life stage (egg, larva, pupa) or the adult life stage, for cardinal aspect (**A**, **B**), and vegetation type (**C**, **D**). Horizontal dashed lines indicate weak, moderate, and strong associations to aid interpretation. All data points are shown; significant values are denoted with triangles, non-significant values are denoted with circles. Lines connect datapoints within foodplant–Lepidoptera species pairs

At the reserve-scale, there was no significant difference between habitat specialists and generalists in the strength of their associations with aspect (W = 15, p = 0.383; Fig. S1A, B) or vegetation type (W = 16, p = 0.267; Fig. S1C, D). There was a significant difference in the strength of association between Lepidoptera that overwinter at the adult life stage compared to species that overwinter at non-adult life stages for aspect (W = 2, p = 0.024) (Fig. 5A, B), whereby species that overwinter at non-adult life stages had stronger associations with aspect. There was no significant difference in association for vegetation type (W = 10, p = 0.527) (Fig. 5C, D).

At the foodplant patch scale, there was no significant difference in the strength of association between habitat specialists and generalists with plant height (W = 10, p = 0.571) or plant density (W = 6, p = 0.786) (Fig. S2A, B). There was also no significant difference in the strength of association between Lepidoptera that overwinter at the adult life stage compared to species that overwinter at the non-adult life stages with plant height (W = 9, p = 0.905) or plant density (W = 11, p = 0.905) (Fig. S2C, D). There were no obvious patterns in association between the two scales.

## Discussion

Day-flying Lepidoptera and their foodplants showed varied associations with habitat characteristics across the reserve and foodplant scale. At the reserve scale, across all 11 species, habitat associations for both Lepidoptera and their foodplants were generally stronger with vegetation type than aspect, although six species maintained associations with aspect and five with vegetation type when compared to their foodplant distributions. *E. tages* and *P. coridon* were associated with south-facing slopes, *A. io* and *M. jurtina* with flat areas, and *A. urticae* and *A. cardamines* with north-facing slopes. *C. minimus*, *A. io,* and *A. cardamines* were associated with encroaching scrub, *A. urticae* with long grass, and *P. coridon* with short grass. In general, ecological traits did not influence the strength of association with habitats at the reserve scale, except for overwintering life stage, whereby species that overwinter at non-adult life stages had stronger larval associations with aspect than species that overwinter at the adult life stage. At the foodplant scale, five species showed no significant effect of foodplant characteristics (plant height and density) on larval presence, and four species showed significant effects. *C. minimus* and *A. cardamines* were more likely to be present on taller foodplants, while *A. io* and *A. urticae* were more likely to be present in denser foodplant patches. There were no trends in these associations with the ecological traits; habitat specialism or overwintering life stage. There were no consistent patterns of association across the two scales investigated in this study.

At the reserve scale, Lepidoptera and their foodplants showed similar weak to moderate associations with aspect. For plants, aspect is likely to influence resource availability, in the form of soil water (Packepsky et al. 2001), nutrient availability (Garten et al. 1994), and local temperature conditions (Bennie et al. 2008). Of the foodplants tested, all bar one had a significant association with aspect, the exception being *R. cathartica*. This shrub species is highly tolerant of variable environmental conditions, for example being found in both dry and flooded soils (Knight et al. 2007). This resilience may explain the lack of association with particular aspects. An important caveat to consider is that the data from this study comes from just four nature reserves in Bedfordshire, UK, and were collected over just 2 years. Therefore the reserve scale associations detected may not reflect habitat associations in other parts of species’ ranges, or associations that could be detected over longer time-periods or more variable climatic conditions. However, the sites selected were chosen because of their high topographic and vegetation variation, and therefore the results should be relatively robust.

Of the 11 Lepidoptera species tested, ten had significant associations with aspect, six of which were maintained when compared to their foodplant distributions (*E. tages*, *P. coridon*, *A. io*, *A. urticae*, *M. jurtina*, *A. cardamines*). This implies that the associations detected are true associations of the larvae, rather than being driven by foodplant distribution. This group includes species that are restricted habitat specialists and very generalist species. Associations with aspect have been identified in Lepidoptera species before (e.g. Davies et al. 2006; Anthes et al. 2008), and are often attributed to the thermal benefits that these aspects provide. Temperature drives many physiological processes in ectotherms relevant to fitness (Kingsolver et al. 2013). For Lepidoptera larvae, higher temperatures are associated with increased growth and development rates (Moallem et al. 2017), but excessively high temperatures can result in death (Chown & Jaco Klok 1997). Of the six species with associations with aspect, two had associations with north-facing (and therefore cooler and damper) aspects (*A. urticae*, *A. cardamines*), two species with south-facing (and therefore warmer and drier) aspects (*E. tages*, *P. coridon*), and two species with flat land (*A. io*, *M. jurtina*). The species associated with cooler aspects may be at particular risk under future climate change, as these aspects will warm and there are no other cooler aspects to exploit. A possible response to this could be for species to change their phenology within their current distribution, or to shift distribution towards cooler latitudes. Contrastingly, species that rely on warmer aspects now may find that there is more suitable habitat as the climate warms, and they can expand from south-facing slopes to other aspects (Thomas et al. 2001), should this association be plastic. However, if this association with warmer aspects is fixed, it may become a maladaptive trait in the future (Salgado et al. 2020), with consequences for the persistence of these species under climate change. Previous studies have detected shifts in associations with habitat characteristics over time (Davies et al. 2006), whereas others have found consistent associations (Hayes et al. 2021; Ashe-Jepson et al. 2022). It is important to note that we were only able to assess habitat characteristic associations for 11 species in this study, and it is possible that trends could be more or less marked when more UK Lepidoptera are taken into account. Further research is needed to determine whether associations with aspect are plastic or fixed, and to assess this across a wider range of species. Nevertheless, the range of associations we detected highlights how reserves with varied topographies are likely to be essential for supporting a range of species. Previous studies have found links between topographic diversity and increased butterfly population stability at larger spatial scales than used in this study (Oliver et al. 2010; Suggitt et al. 2015). The fact that a similar trend has been detected at the more local reserve scale further demonstrates the high value of topographic variation for conservation.

Lepidoptera and their foodplants generally showed stronger associations with vegetation type than aspect at the reserve scale. The vegetation type categories used in this study are broad, and are intended to reflect plant communities. It is, therefore, not a surprise that foodplants and associated Lepidoptera have strong associations with vegetation types. However, the variation in associations with vegetation type across species suggests that reserves with varied vegetation structure will support a more diverse butterfly community. Five of the species tested showed associations with vegetation type that were maintained when compared to foodplant associations. Three of these were associated with encroaching scrub (*C. minimus*, *A. io*, *A. cardamines*), the most sheltered vegetation category, one with long grass (*A. urticae*), and one with short grass (*P. coridon*), the most exposed vegetation category. These associations may reflect a benefit that different vegetation types confer to the larvae, such as encroaching scrub providing protection from predators (Atkinson et al. 2004) or shelter from wind (Bauerfeind et al. 2009). Vegetation structure also alters temperature conditions (Green et al. 1984), resulting in different microclimates that larvae will experience (Curtis & Isaac 2015). However, across all species tested, we detected a general decrease in the strength of association of Lepidoptera with vegetation type when compared to foodplant distributions. This implies that vegetation associations are driven, at least in part, by foodplant distributions and, therefore, that there is some flexibility in larval associations with vegetation types. If so, landscapes may be suitable for Lepidoptera across a wider range of broad categories, as long as foodplants are present. However, this flexibility is not reflected in the foodplants. Therefore management for these Lepidoptera species should focus on providing suitable vegetation types for the foodplants, which can be altered or maintained through techniques such as grazing (Carmel & Kadmon 1999) or cutting (Parr & Way 1988).

At the reserve scale, we found limited evidence that species classified at the national scale as habitat specialists had stronger habitat associations than habitat generalists. However, it is important to note again that the limited number of species investigated may have influenced our ability to detect differences between these groups. We call for more research to investigate differences in habitat associations between habitat generalists and specialists across a wider range of species, and with greater sample sizes. Habitat specialism is also context dependent, and therefore some species that are classified as habitat generalists nationally may be acting as specialists at more local scales, obscuring any patterns between groups. As such, future research should investigate associations with habitat characteristics across a wider range of habitat types. In general, there were weak trends for habitat specialist species to have stronger associations with both aspect and vegetation type than habitat generalists, particularly when considering their foodplant distributions. In particular, there appears to be a marked weakening of association for habitat generalists with vegetation type when considering their foodplant distributions. This implies that vegetation type management for habitat generalist species could focus on providing suitable habitat for their foodplants, which in turn will benefit the Lepidoptera. Overall, the lack of difference between these groups implies that topographic and vegetation variation is equally important for both specialists and generalists, and therefore conserving sites with high heterogeneity in both of these characteristics will benefit a wide variety of species.

Lepidoptera species that overwinter at non-adult life stages (egg, larva, pupa) had stronger reserve scale associations with aspect than species that overwinter as adults, but not with vegetation type. Aspect and vegetation type are both likely to alter local microclimate through winter, and associations with these factors likely reflects the importance of the thermal benefits they provide. For example, aspect influences the amount of solar radiation received; as solar radiation is highest on south-facing slopes in the northern hemisphere (Rorison et al. 1986), this would translate to relatively warmer and drier conditions (Bennie et al. 2006). In Lepidoptera, the life stage at which a species overwinters is particularly vulnerable to adverse conditions, such as extreme temperatures (McDermott Long et al. 2017; Abarca et al. 2019). Therefore, there could be strong pressure to select and occupy habitats that maximise chances of survival for species overwintering at a non-adult life stage. For these less mobile life stages, available overwintering locations are likely to be in the immediate area surrounding their foodplant (Tjørnløv et al. 2015), and therefore the strong habitat associations we observed may reflect adaptations to select areas that are suitable for overwintering as well as larval growth and development. This requirement could result in these species having more restricted choices when selecting habitats for their offspring, potentially rendering them more vulnerable to environmental change. The stronger association we found with aspect rather than vegetation type may reflect a greater variation in vegetation characteristics across seasons, whereby aspect may be a more reliable indicator of microclimatic conditions over winter, which could be exploited by egg-laying females. For example, *P. coridon* overwinters as an egg, a particularly vulnerable and sessile life stage whose distribution is entirely dependent on the female’s selection of oviposition sites. This species had one of the strongest associations with aspect, preferentially occupying south-facing slopes, which are warmer and drier (Bennie et al. 2006). From a management perspective, aspect can rarely be manipulated (although see Hayes et al*. in review*). Instead, aspect variation should be considered when selecting land to prioritize for protection. We are not aware of any studies that have investigated aspect preferences in overwintering eggs, larvae or pupae, making this a priority for future work.

By contrast, species that overwinter as adults are more mobile, and able to search across landscapes, away from their natal foodplant, for suitable overwintering locations (Dvořák et al. 2009). We found that species that overwinter as adults had weak associations with aspect and vegetation type compared to their foodplant distribution. However, their foodplants had strong associations with vegetation type, which implies that management for these species should focus on providing suitable vegetation characteristics for their foodplants.

When considering habitat associations of Lepidoptera, the amount of suitable habitat is often constrained by the associations of their foodplants. In general, the Lepidoptera species we tested had similar reserve scale habitat associations to their foodplants. This implies that land which is suitable for foodplants is also suitable for Lepidoptera, and management that confers benefits to foodplants will also benefit Lepidoptera. However, there were four notable exceptions; *E. tages*, *C. minimus*, *P. coridon,* and *A. urticae*. The larvae of *E. tages* were associated with south-facing slopes, reflecting a preference identified in other studies (Dickins et al. 2013), whereas their foodplant (*L. corniculatus*) was associated with west-facing slopes. This indicates that land with the foodplant present which includes both south-facing and west-facing slopes for may be the most valuable for this species. The underlying causes behind these associations are unknown, but may reflect a requirement for the thermal benefits a south-facing slope in the UK would provide. This species is spring-flying as an adult, completing larval development in April, and overwintering as larvae (Asher et al. 2001). As a result, *E. tages* may be occupying south-facing slopes as they provide warm microclimates to speed up larval and pupal development during spring, and may contribute to enhanced overwinter survival.

Another species with divergent habitat associations compared to its foodplant was *C. minimus*, where the larvae were associated with encroaching scrub, whereas the foodplant (*Anthyllis vulneraria*) was associated with short grass. *C. minimus* may benefit from the shelter that scrub provides, for example protection from predators (Atkinson et al. 2004), by altering the temperature and humidity conditions (Turlure et al. 2011), or alternatively that the soil under scrub has higher nutrient content (Gough & Marrs 1990), which may benefit foodplant growth, and provide increased resources for the seed-feeding larvae. Our findings suggest that management for this species should incorporate both short grass and encroaching scrub, which supports ongoing conservation management practice for this species. Management of grasslands often includes scrub clearance (Redhead et al. 2012), but this association implies that scrub edges may provide suitable habitat for this rare, localised, and declining species in the UK (Fox et al. 2023), and that some scrub should be maintained, particularly near areas with high foodplant abundance.

The third species where Lepidoptera and their foodplant had differing reserve scale habitat associations was *A. urticae*, one of three nettle-feeding species in this study. *A. urticae* larvae were associated with long grass, whereas their foodplant (*Urtica dioica*) was associated with encroaching scrub. Long grass vegetation types are less sheltered than encroaching scrub, and should receive more direct sunlight. Sunlight is an oviposition cue in some nymphalid butterflies (Braem & Van Dyck 2021), but to our knowledge this has not been recorded in this species. Alternatively, *A. urticae* may be selecting more open habitats due to the thermal benefits this provides. Receiving more direct sunlight may confer benefits to larvae, with direct effects such as increasing growth rate, or indirect effects such as impacts on their foodplant or natural enemies (Battisti et al. 2013). A previous study found that direct sunlight reduced the development time of *A. urticae* larvae by 20%, which was attributed to higher temperatures (Bryant et al. 2002). This preference for more exposed habitats may explain why *A. urticae* is one of the most northerly distributed of the nettle-feeding nymphalids in the UK (Bryant et al. 2003), and supports known patterns of behaviour in this species to oviposit on leaves in full sunlight (Asher et al. 2001). However, we also detected a preference for north-facing slopes for *A. urticae*, which implies that it was not simply selecting the hottest habitats available. This is also in contrast to their foodplant, which favoured flat land. The foodplant generally grows in sheltered nutrient-rich moist soils (Taylor 2009), and may not perform well when growing in exposed areas receiving direct sunlight in calcareous grasslands, which tend to have free-draining, low nutrient soils (Jamieson et al. 1998). *U. dioica* growing on north-facing slopes will be growing in soils with greater water content (Reid 1973), which could confer benefits to the larvae, such as increasing larval growth and survival (Pullin 1987; Pollard et al. 1997). The combination of associating with open exposed vegetation types and cooler, damper aspects results in restrictive habitat requirements for *A. urticae*, and could impose restrictions on foodplants suitable for reproduction, although the wide distribution of *U. dioica* makes this unlikely to negatively impact *A. urticae*. However, it is possible that these more restrictive requirements could amplify the effects of other drivers of loss. Indeed, *A. urticae* has undergone one of the largest declines in abundance of any butterfly species in the UK over the last 40 years (− 79%) (Fox et al. 2023).

Alternatively, the habitat associations of *A. urticae* may be the result of niche partitioning. The foodplant of *A. urticae* is shared with other ecologically similar species, particularly *A. io* (Asher et al. 2001). Both these species have gregarious larvae, meaning that foodplant patches could contain exceptionally high larval densities, which would risk defoliating all available foodplants and result in larval starvation. We found divergent habitat associations between these species, with *A. io* larvae associated with flat areas and encroaching scrub, but *A. urticae* favouring north-facing slopes and long grass. This difference in habitat associations could reduce the number of eggs being laid within the same foodplant patches, and allow for the persistence of these similar species within the same landscape. If this is the case, land management for nettle-feeding nymphalids should incorporate varied aspects and vegetation types to support the diverse needs of these species. Further study is needed to determine whether these habitat associations are maintained when these two species occur allopatrically.

At the foodplant scale, we found that four of the nine species tested had significant associations with foodplant characteristics. There were no obvious patterns across ecological traits, possibly due to the species-specific requirements resulting in unique patterns that are not captured within our broad categories, or the restricted number of species included in this study. For example, the two species associated with tall foodplants (*C. minimus*, *A. cardamines*) are the only seed-feeding species in this study (Asher et al. 2001). As seed-feeders, these species have limited food availability on a plant compared to leaf-eating species, which could result in greater selective pressure for egg-laying females to select foodplants that provide sufficient resources for larvae. The two species that were associated with dense patches of foodplants (*A. io*, *A. urticae*) are both nettle-feeding, gregarious species. As such, both are likely to require a large volume of foodplant in a small area, potentially resulting in strong selective pressure for egg-laying females to oviposit in nettle patches that are large enough to sustain large numbers of larvae, and with foodplants close enough to allow larvae to move between plants. The consequence of restrictive preferences for foodplant characteristics means that only a small proportion of available foodplants are used. This implies that selective species may be at greater risk under future environmental change, as climate change is predicted to alter plant communities and characteristics, including plant abundance, phenology, and nutrient content (Cornelissen 2011). At present, it is unclear how species will respond to climate change, and whether these changes will reduce or expand limited foodplant resources. This uncertainty highlights the importance of management to maximise the number of foodplants with diverse traits, and support diverse butterfly communities.

In general, we found limited evidence that patterns of association at the reserve scale were reflected at the foodplant patch scale, though this may have been restricted by sample sizes and the traits recorded. This implies that Lepidoptera species have differing requirements across scales, and means that management for Lepidoptera will require specific strategies at each scale. It also highlights the value of incorporating multiple spatial scales into research on habitat associations. With an understanding of which habitat characteristics are important across differing spatial scales, selection and land management for species conservation can be improved (Orians & Wittenberger 1991; Chalfoun & Martin 2007).

We have highlighted the importance of heterogeneity within nature reserves to support diverse butterfly communities. Maintaining a diverse range of habitat characteristics is also likely to promote climate resilience in insect populations, as this will provide alternative microclimates following environmental change, and help nature reserves to remain valuable and effective under future climate change. To achieve this, there are several routes land-managers could take. It is likely to be more challenging to alter aspect than vegetation type, although artificial landscaping is becoming increasingly common, with ‘butterfly banks’ being built across the UK. These will be particularly valuable within landscapes with little existing topography, however their scale is, at present, relatively limited and so these structures are unlikely to become a successful large-scale solution. Instead, when land is being selected for protection, landscapes containing heterogenous aspects should be considered of higher value than homogenous landscapes. If Lepidopteran associations with aspects reflect thermal requirements, these may be achieved through creating areas of shelter or shade, and/or creating areas of bare ground. Overall, managing nature reserves for aspect heterogeneity will benefit both the foodplants and the insect communities that depend on them. Even minor topographic variation can act as climate refugia for plant species during extreme weather events, such as prolonged drought, and so promotes climate resilience at small spatial scales (Godfree et al. 2011). Secondly, land-managers should promote and maintain a variety of vegetation types. This can be achieved by techniques such as grazing or cutting, while restricting the movement of grazers to allow areas to develop vegetation of different heights. This can be more challenging with wild grazers, such as rabbits, however exclosures can reduce rabbit grazing successfully to the benefit of insect communities (Grayson & Hassall 1985). Scrub clearance, with the maintenance of some scrub patches and scrub-grassland edges, is a common conservation management practice to maintain grasslands (Ellis et al. 2012).. Our results also indicate that scrub edges provide important habitat for multiple species (*C. minimus*, *A. io*, *A. cardamines*), supporting such existing practices.

## Supplementary Information

Below is the link to the electronic supplementary material.

Supplementary file1 (DOCX 12250 KB)

## Data Availability

The data and associated code can be found at: 10.5281/zenodo.18223776
