# Supplementary material for: Habitat associations of day-flying Lepidoptera and their foodplants within nature reserves in Bedfordshire, UK
Source: J Insect Conserv. 2024 Feb 17;28(5):891–908. doi: 10.1007/s10841-024-00554-7 (PMC11489185; doi:10.1007/s10841-024-00554-7)
Supplement: Supplementary file 1 — Supplementary file1 (DOCX 12250 KB) [file 10841_2024_554_MOESM1_ESM.docx]

**Habitat association of butterflies and their foodplants within nature reserves in Bedfordshire, UK**

**Supplementary Materials**

Table S1: Ecological traits of the 11 species of day-flying Lepidoptera used in this study (from Asher *et al.* 2001).

| **Species** | **Family** | **Habitat specialism** | **Overwintering life stage** | **Voltinism** |
| --- | --- | --- | --- | --- |
| *Erynnis tages* | Hesperiidae | Specialist | Larva | Multivoltine |
| *Cupido minimus* | Lycaenidae | Specialist | Larva | Multivoltine |
| *Polyommatus coridon* | Lycaenidae | Specialist | Egg | Univoltine |
| *Aglais io* | Nymphalidae | Generalist | Adult | Univoltine |
| *Aglais urticae* | Nymphalidae | Generalist | Adult | Multivoltine |
| *Maniola jurtina* | Nymphalidae | Generalist | Larva | Univoltine |
| *Vanessa atalanta* | Nymphalidae | Migrant | Adult | Univoltine |
| *Anthocharis cardamines* | Pieridae | Generalist | Pupa | Univoltine |
| *Gonepteryx rhamni* | Pieridae | Generalist | Adult | Univoltine |
| *Pieris napi* | Pieridae | Generalist | Pupa | Multivoltine |
| *Zygaena filipendulae* | Zygaenidae | Generalist | Larva | Univoltine |

Table S2: Explanatory variables (plant height, vegetation height, and plant density) tested for multicollinearity before fitting conditional logistic models. All tests were conducted within species (ordered alphabetically by family and species). As a result of high correlations between plant height and vegetation height (p-values < 0.05 and correlation coefficients >0.7 (Dormann *et al.* 2013), in bold), vegetation height was excluded from subsequent analyses.

| **Terms** | **Correlation coefficient** | **P-value** |
| --- | --- | --- |
| *Erynnis tages* | | |
| Plant height ~ vegetation height | **0.801** | **<0.001** |
| Plant height ~ plant density | -0.215 | 0.182 |
| Vegetation height ~ plant density | -0.085 | 0.602 |
| *Cupido minimus* | | |
| Plant height ~ vegetation height | **0.805** | **<0.001** |
| Plant height ~ plant density | 0.143 | 0.180 |
| Vegetation height ~ plant density | 0.080 | 0.456 |
| *Polyommatus coridon* | | |
| Plant height ~ vegetation height | -0.199 | 0.280 |
| Plant height ~ plant density | -0.212 | 0.243 |
| Vegetation height ~ plant density | 0.248 | 0.171 |
| *Aglais io* | | |
| Plant height ~ vegetation height | **0.717** | **<0.001** |
| Plant height ~ plant density | 0.371 | <0.001 |
| Vegetation height ~ plant density | 0.483 | <0.001 |
| *Aglais urticae* | | |
| Plant height ~ vegetation height | **0.775** | **<0.001** |
| Plant height ~ plant density | 0.178 | 0.045 |
| Vegetation height ~ plant density | 0.175 | 0.052 |
| *Vanessa atalanta* | | |
| Plant height ~ vegetation height | 0.390 | 0.059 |
| Plant height ~ plant density | 0.308 | 0.163 |
| Vegetation height ~ plant density | -0.156 | 0.488 |
| *Anthocharis cardamines* | | |
| Plant height ~ vegetation height | 0.643 | <0.001 |
| Plant height ~ plant density | 0.010 | 0.891 |
| Vegetation height ~ plant density | -0.053 | 0.478 |
| *Gonepteryx rhamni* | | |
| Plant height ~ vegetation height | 0.053 | 0.598 |
| Plant height ~ plant density | -0.315 | 0.001 |
| Vegetation height ~ plant density | -0.156 | 0.120 |
| *Pieris napi* | | |
| Plant height ~ vegetation height | -0.028 | 0.852 |
| Plant height ~ plant density | 0.302 | 0.039 |
| Vegetation height ~ plant density | -0.303 | 0.038 |

Table S3: The results of Chi Square tests and associated Cramer’s V between Lepidoptera and their habitat associations with reserve-scale environmental characteristics: aspect (North, East, South, West, or Flat if the slope is <10°), and vegetation type (short grass (<10 cm), long grass (>10 cm), or encroaching scrub (25-75% of the surrounding 5 m^2^ contains scrub). Three tests were run per species*; the Foodplant (observed distribution of the foodplant with the proportional habitat availability across the sites surveyed used to calculate expected frequencies), the Larva (observed distribution of the larva with the proportional habitat availability across the sites surveyed used to calculate expected frequencies), and the Larva*Foodplant (observed distribution of the larva with the proportional foodplant distribution per habitat type used to calculate the expected frequency). Where counts were too low for analysis, NAs are given. Significant associations are in **bold**.

* *Maniola jurtina* had only one test run (Larva distributions), as they feed on grass and the distribution of their foodplants could not be mapped.

|  | **Aspect** | | | **Vegetation type** | | |
| --- | --- | --- | --- | --- | --- | --- |
|  | **Chi sq** | **P-value** | **Cramer's V** | **Chi sq** | **P-value** | **Cramer's V** |
| *Erynnis tages* | | | | | | |
| Foodplant | 1959.700 | **<0.001** | 0.291 | 185.710 | **<0.001** | 0.285 |
| Larva | 95.141 | **<0.001** | 0.265 | 18.919 | **<0.001** | 0.376 |
| Larva * foodplant | 15.136 | **0.004** | 0.183 | 5.289 | 0.072 | 0.210 |
| *Cupido minimus* | | | | | | |
| Foodplant | 498.440 | **<0.001** | 0.152 | 247.830 | **<0.001** | 0.395 |
| Larva | 60.182 | **<0.001** | 0.185 | 0.100 | 0.945 | 0.031 |
| Larva * foodplant | 8.212 | 0.082 | 0.115 | 34.275 | **<0.001** | 0.446 |
| *Polyommatus coridon* | | | | | | |
| Foodplant | 93.875 | **<0.001** | 0.289 | 21.711 | **<0.001** | 0.442 |
| Larva | 169.780 | **<0.001** | 0.345 | 67.569 | **<0.001** | 0.693 |
| Larva * foodplant | 65.729 | **<0.001** | 0.267 | 32.727 | **<0.001** | 0.188 |
| *Aglais io* | | | | | | |
| Foodplant | 210.620 | **<0.001** | 0.050 | 2716.100 | **<0.001** | 0.414 |
| Larva | 217.220 | **<0.001** | 0.124 | 442.450 | **<0.001** | 0.406 |
| Larva * foodplant | 74.313 | **<0.001** | 0.057 | 22.948 | **<0.001** | 0.092 |
| *Aglais urticae* | | | | | | |
| Foodplant | 210.620 | **<0.001** | 0.050 | 2716.100 | **<0.001** | 0.414 |
| Larva | 78.400 | **<0.001** | 0.100 | 145.790 | **<0.001** | 0.314 |
| Larva * foodplant | 99.014 | **<0.001** | 0.089 | 636.030 | **<0.001** | 0.651 |
| *Vanessa atalanta* | | | | | | |
| Foodplant | 210.620 | **<0.001** | 0.050 | 2716.100 | **<0.001** | 0.414 |
| Larva | 2.975 | 0.562 | 0.077 | 26.639 | **<0.001** | 0.530 |
| Larva * foodplant | 1.155 | 0.910 | 0.038 | 1.137 | 0.607 | 0.109 |
| *Maniola jurtina* | | | | | | |
| Larva | 15.938 | **0.008** | 0.139 | 4.634 | 0.100 | 0.238 |
| *Anthocharis cardamines* | | | | | | |
| Foodplant | 395.190 | **<0.001** | 0.042 | 3675.800 | **<0.001** | 0.359 |
| Larva | 13.866 | **0.012** | 0.031 | 144.240 | **<0.001** | 0.288 |
| Larva * foodplant | 20.232 | **<0.001** | 0.276 | 79.653 | **<0.001** | 0.150 |
| *Gonepteryx rhamni* | | | | | | |
| Foodplant | 5.098 | 0.270 | 0.030 | 46.534 | **<0.001** | 0.420 |
| Larva | 12.246 | **0.020** | 0.060 | 72.256 | **<0.001** | 0.461 |
| Larva * foodplant | 7.649 | 0.101 | 0.036 | 0.867 | 0.386 | 0.058 |
| *Pieris napi* | | | | | | |
| Foodplant | 14.468 | **0.015** | 0.049 | 96.461 | **<0.001** | 0.372 |
| Larva | 13.694 | **0.018** | 0.062 | 60.821 | **<0.001** | 0.381 |
| Larva * foodplant | 0.715 | 0.740 | 0.040 | 0.074 | 1.000 | 0.019 |
| *Zygaena filipendulae* | | | | | | |
| Foodplant | 1656.700 | **<0.001** | 0.325 | 118.290 | **<0.001** | 0.277 |
| Larva | 28.741 | **<0.001** | 0.269 | 1.425 | 0.553 | 0.191 |
| Larva * foodplant | 5.149 | 0.263 | 0.199 | 0.671 | 0.761 | 0.136 |

Table S4: The results of conditional logistic regressions between larval presence and foodplant characteristics (foodplant height (cm) and foodplant density (either as a count of individual foodplants or, in cases where foodplants were non-distinct and overlapping, as percentage cover within 30 cm of the foodplant). Tests were run individually by species (ordered alphabetically by family and species). Species sampled by suction sampler (*Maniola jurtina, Zygaena filipendulae)* were excluded from this analysis, as it was not possible to determine which specific foodplant they occupied before capture.

| **Term** | **Coefficient** | **Exponentiated coefficient** | **Standard error** | **Lower 95% C.I.** | **Upper 95% C.I.** | **P-value** |
| --- | --- | --- | --- | --- | --- | --- |
| *Erynnis tages* | | | | | | |
| Foodplant height | -0.217 | 0.805 | 0.255 | 0.488 | 1.328 | 0.396 |
| Foodplant density | 0.029 | 1.029 | 0.042 | 0.948 | 1.117 | 0.497 |
| *Cupido minimus* | | | | | | |
| Foodplant height | 0.085 | 1.089 | 0.038 | 1.011 | 1.174 | **0.025** |
| Foodplant density | 0.072 | 1.075 | 0.039 | 0.996 | 1.16 | 0.064 |
| *Polyommatus coridon* | | | | | | |
| Foodplant height | 0.108 | 1.114 | 0.067 | 0.977 | 1.27 | 0.106 |
| Foodplant density | -0.053 | 0.948 | 0.642 | 0.269 | 3.337 | 0.934 |
| *Aglais io* | | | | | | |
| Foodplant height | 0.008 | 1.008 | 0.01 | 0.989 | 1.027 | 0.409 |
| Foodplant density | 0.045 | 1.046 | 0.011 | 1.023 | 1.069 | **<0.001** |
| *Aglais urticae* | | | | | | |
| Foodplant height | -0.039 | 0.962 | 0.02 | 0.924 | 1.001 | 0.054 |
| Foodplant density | 0.053 | 1.054 | 0.022 | 1.01 | 1.1 | **0.015** |
| *Vanessa atalanta* | | | | | | |
| Foodplant height | 0.067 | 1.069 | 0.045 | 0.979 | 1.168 | 0.138 |
| Foodplant density | 0.034 | 1.034 | 0.036 | 0.965 | 1.109 | 0.342 |
| *Anthocharis cardamines* | | | | | | |
| Foodplant height | 0.031 | 1.032 | 0.008 | 1.016 | 1.048 | **<0.001** |
| Foodplant density | -0.038 | 0.963 | 0.072 | 0.836 | 1.109 | 0.601 |
| *Gonepteryx rhamni* | | | | | | |
| Foodplant height | -0.011 | 0.989 | 0.012 | 0.966 | 1.013 | 0.363 |
| Foodplant density | 21.01 | 1.34E+09 | 24380 | 0 | Inf | 0.999 |
| *Pieris napi* | | | | | | |
| Foodplant height | -0.046 | 0.955 | 0.116 | 0.761 | 1.198 | 0.689 |
| Foodplant density | 0.089 | 1.093 | 0.134 | 0.841 | 1.42 | 0.507 |


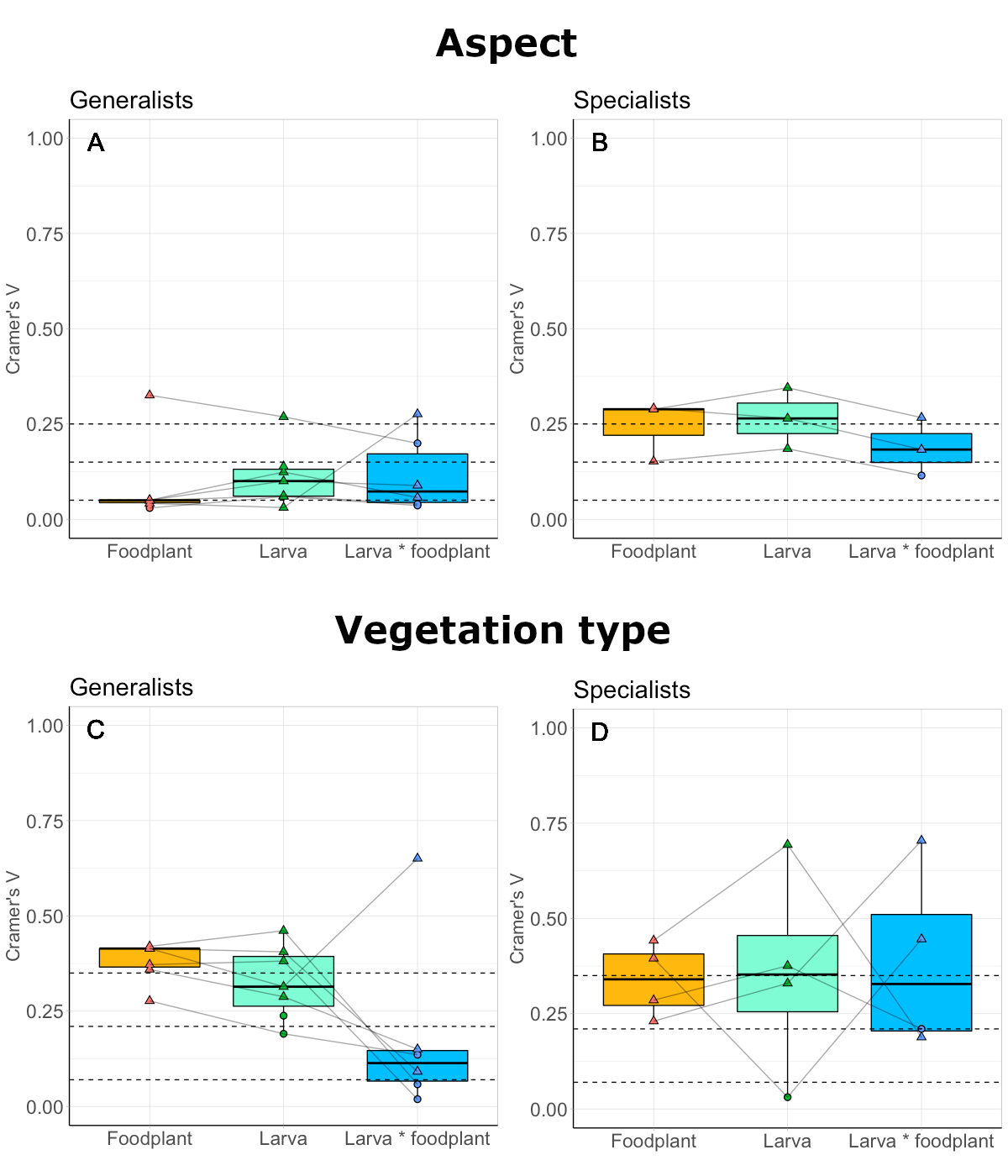


Figure S1: The strength of association (Cramer’s V) of foodplants (Foodplant) and larvae (Larva) against the total available habitat area, and of larvae against the foodplant distribution (Larva * foodplant), for habitat generalists and specialists for cardinal aspect (A, B), and vegetation type (C, D). Horizontal dashed lines indicate weak, moderate, and strong associations to aid interpretation, calculated from the degrees of freedom. All data points are shown; significant values are denoted with triangles, non-significant values are denoted with circles. Lines connect datapoints within foodplant-Lepidoptera species pairs.


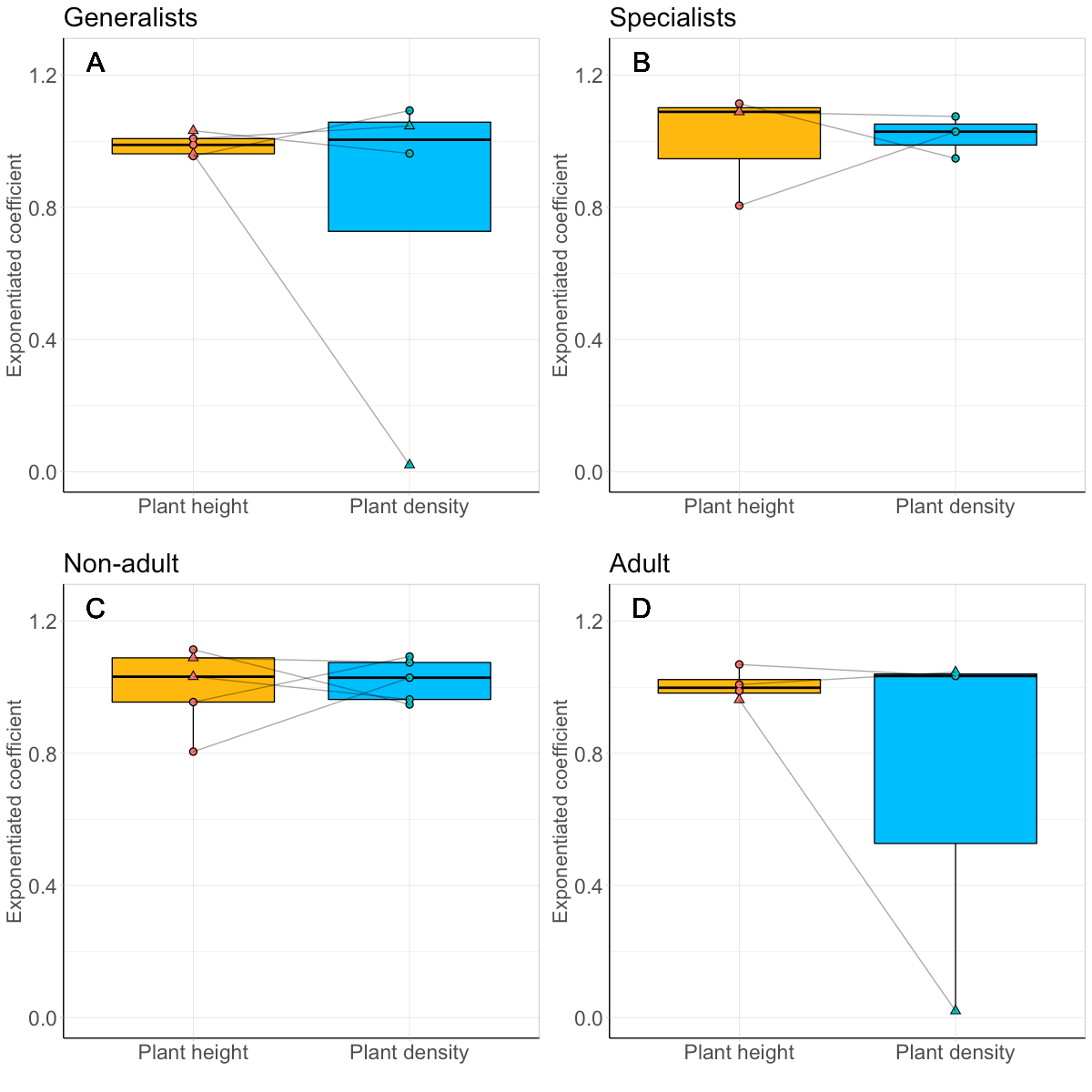


Figure S2: The strength of association (exponentiated coefficients from conditional logistic regressions) of larval presence against foodplant characteristics, foodplant height (cm), and foodplant density (either as individual counts or percentage cover within 30 cm). All data points are shown; significant values are denoted with triangles, non-significant values are denoted with circles. Lines connect datapoints of the same species.

**References**

Asher, J., Warren, M., Fox, R., Harding, P., Jeffcoate, G. & Jeffcoate, S. (2001). The millennium atlas of butterflies in Britain and Ireland. *Millenn. atlas butterflies Britain Ireland.*

Dormann, C.F., Elith, J., Bacher, S., Buchmann, C., Carl, G., Carré, G., *et al.* (2013). Collinearity: A review of methods to deal with it and a simulation study evaluating their performance. *Ecography (Cop.).*, 36, 27–46.

Smart, J., Sutherland, W.J., Watkinson, A.R. & Gill, J.A. (2004). A New Means of Presenting the Results of Logistic Regression. *Bull. Ecol. Soc. Am.*, 85, 100–102.

Stubben, C.J. & Milligan, B.G. (2007). Estimating and Analyzing Demographic Models Using the popbio Package in R. *J. Stat. Softw.*, 22.
